# Conjugates of Ciprofloxacin and Levofloxacin with Cell-Penetrating Peptide Exhibit Antifungal Activity and Mammalian Cytotoxicity

**DOI:** 10.3390/ijms21134696

**Published:** 2020-06-30

**Authors:** Natalia Ptaszyńska, Katarzyna Gucwa, Katarzyna Olkiewicz, Mateusz Heldt, Marcin Serocki, Anna Stupak, Dorota Martynow, Dawid Dębowski, Agata Gitlin-Domagalska, Jan Lica, Anna Łęgowska, Sławomir Milewski, Krzysztof Rolka

**Affiliations:** 1 Department of Molecular Biochemistry, Faculty of Chemistry, University of Gdansk, 80-308 Gdańsk, Poland; natalia.ptaszynska@ug.edu.pl (N.P.); katarzynach3@wp.pl (K.G.); katarzyna.olkiewicz@phdstud.ug.edu.pl (K.O.); dawid.debowski@ug.edu.pl (D.D.); agata.domagalska@ug.edu.pl (A.G.-D.); anna.legowska@ug.edu.pl (A.Ł.); krzysztof.rolka@ug.edu.pl (K.R.); 2 Department of Pharmaceutical Technology and Biochemistry, Faculty of Chemistry, Gdansk University of Technology, 80-233 Gdańsk, Poland; mateusz.heldt@pg.edu.pl (M.H.); marcin.serocki@pg.edu.pl (M.S.); dorota.koperkiewicz@gmail.com (D.M.); slamilew@pg.edu.pl (S.M.); 3 Laboratory of Bacterial Genetics, Faculty of Chemistry, Gdansk University of Technology, 80-233 Gdańsk, Poland; anna.stupak1@pg.edu.pl

**Keywords:** cell-penetrating peptide (CPP), transportan 10 (TP10-NH_2_), ciprofloxacin bioconjugates, levofloxacin bioconjugates, antimicrobial agents, bacteriostatic and mycostatic agents, redox-resistant and redox-sensitive linkers

## Abstract

Seven conjugates composed of well-known fluoroquinolone antibacterial agents, ciprofloxacin (CIP) or levofloxacin (LVX), and a cell-penetrating peptide transportan 10 (TP10-NH_2_) were synthesised. The drugs were covalently bound to the peptide via an amide bond, methylenecarbonyl moiety, or a disulfide bridge. Conjugation of fluoroquinolones to TP10-NH_2_ resulted in congeners demonstrating antifungal in vitro activity against human pathogenic yeasts of the *Candida* genus (MICs in the 6.25–100 µM range), whereas the components were poorly active. The antibacterial in vitro activity of most of the conjugates was lower than the activity of CIP or LVX, but the antibacterial effect of CIP-S-S-TP10-NH_2_ was similar to the mother fluoroquinolone. Additionally, for two representative CIP and LVX conjugates, a rapid bactericidal effect was shown. Compared to fluoroquinolones, TP10-NH_2_ and the majority of its conjugates generated a relatively low level of reactive oxygen species (ROS) in human embryonic kidney cells (HEK293) and human myeloid leukemia cells (HL-60). The conjugates exhibited cytotoxicity against three cell lines, HEK293, HepG2 (human liver cancer cell line), and LLC-PK1 (old male pig kidney cells), with IC_50_ values in the 10–100 µM range and hemolytic activity. The mammalian toxicity was due to the intrinsic cytoplasmic membrane disruption activity of TP10-NH_2_ since fluoroquinolones themselves were not cytotoxic. Nevertheless, the selectivity index values of the conjugates, both for the bacteria and human pathogenic yeasts, remained favourable.

## 1. Introduction

Levofloxacin (LVX) and ciprofloxacin (CIP) are broad-spectrum fluoroquinolone synthetic chemotherapeutics, active against both Gram-positive and Gram-negative bacteria. LVX and CIP are used worldwide to treat a number of bacterial infections, including sinusitis, pneumonia, urinary tract infections, tuberculosis, and meningitis. The antimicrobial spectrum of fluoroquinolones is limited to bacteria and does not include human pathogenic eukaryotic microorganisms [1]. Their mammalian toxicity is generally low. Both are on the WHO List of Essential Medicines containing the most effective and safe medicines needed in the health system [2]. Like all fluoroquinolones, LVX, and CIP function by inhibiting bacterial gyrase and topoisomerase IV [3]. Resistance to fluoroquinolones, which is common in staphylococci, enterococci, and *Pseudomonas* spp., occurs in multiple ways, including alterations in DNA gyrase subunit A, topoisomerase IV, as well as overexpression of multidrug-resistance (MDR) efflux pumps [4].

One of the possibilities to alter the biological properties of chemotherapeutic agents is their chemical modification, including conjugation with oligopeptides, especially those known as the cell-penetrating peptides (CPPs). Such conjugates may possess additional advantages, including an increased selectivity of drug delivery, enhanced efficacy, reduced systemic toxicity, as well as improved pharmacokinetics and pharmacodynamics. Several examples of CPP:drug conjugates have been reported, although most of them concern those of CPPs with anticancer agents [5]. Among very few CPP:antimicrobial agent congeners, those worth mentioning are conjugates of arginine oligomers with triclosan exhibiting promising chemotherapeutic effect in the murine model of toxoplasmosis [6] and peptide-vancomycin conjugates demonstrating enhanced effectiveness against vancomycin-resistant *Acinetobacter baumannii* and *Enterococcus faecalis* [7]. The only example of similar derivatisation of fluoroquinolones was enrofloxacin and ciprofloxacin conjugates with β-octaarginine, showing a similar or lower in vitro antibacterial potential than the mother chemotherapeutics [8]. On the other hand, in our previous studies, we demonstrated some beneficial results of the conjugation of CIP and LVX with antimicrobial peptides (AMPs), namely the lactoferricin truncated analogues [9] and lactoferrin HLopt2 fragment [10].

Cell-penetrating peptides (CPPs), serving as nanocarriers, are considered the fundamental part of a new concept of drug delivery systems [11], which has attacted great interest from many research groups. The unique ability of CPPs to penetrate the plasma membrane makes them a useful tool for the delivery of a vast range of different biologically active compounds to eukaryotic and prokaryotic cells, including proteins, nucleic acids, oligonucleotides, liposomes, nanoparticles, peptides, and low molecular weight chemotherapeutic agents (e.g., doxorubicin, methotrexate, cyclosporine A, paclitaxel [12], and antibiotics [13]). Since the discovery in 1988 of the first cell-penetrating peptide, trans-activator protein (TAT) [14,15], more than 1700 CPPs have been described [16]. One of the well-known CPPs is transportan (TP), a chimeric oligopeptide composed of the first 12 amino acid residues of the neuropeptide galanin and the 14-amino-acid-residue-long wasp venom peptide, mastoparan, connected via a lysine residue [17]. A shorter variant of TP, with deletion of the *N*-terminal hexapeptide, named TP10-NH_2_, having an amide moiety at the *C*-terminus, retains the efficient cell penetration property of the parent compound, with significantly less potential side effects [18]. This peptide amide enters all cell types, including the mammalian ones, and inhibits the growth of some microorganisms, such as *Candida albicans, Staphylococcus aureus* [19], *Streptococcus pneumoniae*, and *Mycobacterium tuberculosis* [20]. TP10-NH_2_ can transport cargo across cell membranes, applying different mechanisms, dependent on the size and character of the vectors delivered [21]. Recently, Rusiecka and co-workers showed that TP10-NH_2_ improves the cytotoxic activity of cisplatin against cancer cell lines. Unconjugated TP10-NH_2_ induces a cytostatic effect on HeLa (cervical cancer) and OS143B (osteosarcoma) cancer cell lines. At the same time, TP10-NH_2_, as well as TP10-NH_2_ in complex with cisplatin, had no impact on the cell viability of two non-cancer cell lines HEK 293 (embryonic kidney) and HEL 299 (lung fibroblasts) [22]. TP10-NH_2_ may thus not only facilitate the transport of an active agent across the microbial cell membranes, but, having an intrinsic antimicrobial activity, it may also potentiate an antimicrobial efficacy of any conjugated antibiotic.

Taking into consideration the literature data mentioned above, we decided to design, synthesise, and determine the antimicrobial activity of conjugates composed of TP10-NH_2_ and levofloxacin or ciprofloxacin. The series of synthesised compounds consisted of five CIP- and two LVX-based conjugates. The conjugation did not improve the antibacterial activity of the mother fluoroquinolones on average. Surprisingly though, they gained activity against human pathogenic yeasts.

## 2. Results and Discussion

### 2.1. Chemical Synthesis and Stability Studies

#### 2.1.1. Synthesis

**Figure 1:**
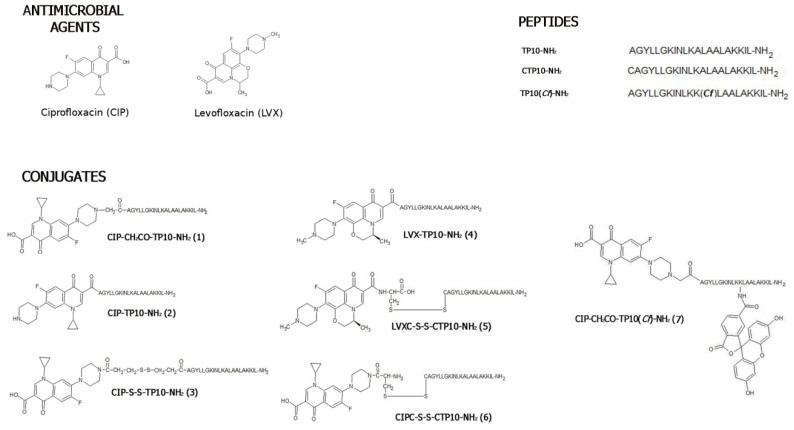
Chemical structures of ciprofloxacin and levofloxacin conjugates with transportan 10 and their constituents.

Seven conjugates composed of TP10-NH_2_ and either levofloxacin or ciprofloxacin were synthesised (Figure 1). In three of them (**3**, **5**, and **6**), the constituents were linked by a labile disulfide bridge. Conjugate **7**, as a fluorescent representative lacking a disulfide bridge, was synthesised solely for cell penetration studies. Data from the HPLC and MS analyses, as well as the total yields of all final compounds, are given in Table S1. In CIP-CH_2_CO-TP10-NH_2_ (**1**), a methylene carbonyl linker was applied to connect the antibacterial agent through its amino functionality to the *N*-terminus of TP10-NH_2_. In CIP-TP10-NH_2_ (**2**), an amide bond was formed between the carboxyl group of CIP and the peptide α-amino group. An intermolecular disulfide bridge, spanning both components in CIP-S-S-TP10-NH_2_ (**3**), was formed using the Lomant’s reagent, dithio-bis(succinimidyl propionate). In the LVX-based conjugate LVX-TP10-NH_2_ (**4**), the LVX carboxyl group was linked with the α-amino group of TP10-NH_2_. In LVXC-S-S-CTP10-NH_2_ (**5**) and CIPC-S-S-CTP10-NH_2_ (**6**), the disulfide bridge was formed by Cys residues, attached to both LVX or CIP and the *N*-terminus of TP10-NH_2_. It was performed by the introduction of the *S*-(3-nitro-2-pyridylsulfanyl) (Npys) group to the thiol function of Cys attached to Boc-CIP or LVX. In order to determine the cellular uptake of **1**, its fluorescently labelled analogue CIP-CH_2_CO-TP10(***Cf***)-NH**_2_** (**7**), TP10(***Cf***)-NH_2_, and CIP(***Cf***) (reference compounds) were also synthesised. In the first two compounds, 5(6)-carboxyfluorescein was attached to the ε-amino group of thte peptide’s Lys residue that replaced Ala in position 12, whereas in CIP(***Cf***), the fluorophore was introduced to the chemotherapeutic amino group.

#### 2.1.2. Stability

In our recent paper [10], we showed that ciprofloxacin conjugated through a disulfide bridge with the antimicrobial peptide named HLopt2 entered microbial cells. After 2 h of incubation, in 2/3 of the conjugate molecules that entered microbial cells, the linker was reduced but remained intact when incubated for 2 h with supernatant obtained from *S. aureus* culture. We obtained similar results for CIP-S-S-TP10-NH_2_ (**3**). Preliminary LC-MS analysis revealed that under the same condition, four peaks were identified. They corresponded to intact conjugate (m/z 2685.56), HS-(CH_2_)_2_CO-TP10-NH_2_ (m/z 2268.41), its dimer (m/z 4534.86), and CIP-CO(CH_2_)_2_-SH (m/z 420.14; [M+H]^+^) (Figure S1). Unlike antimicrobial peptide HLopt2, which was quickly further degraded [10], TP10-NH_2_ was resistant inside the bacterial cells. The identified dimer of HS-(CH_2_)_2_CO-TP10-NH_2_ is a product of peptide oxidation inside the MS spectrometer.

LC-MS analysis of conjugate **3** proved that the disulfide bridge used as a linker between the peptide and antimicrobial is reduced inside the cell, and both components’, CIP and TP10-NH_2_, derivatives can interact with the targets inside the cell. Both are equipped with thiol groups that can also interact in the cytoplasm with glutathione and other compounds containing SH groups, modifying conjugate constituents. In addition, TP10-NH_2_, acting as CPP peptide, helps CIP to pass through the membrane.

CIP-S-S-TP10-NH_2_ was stable in phosphate-buffered saline (PBS) and growth media used for the determination of antibacterial and antifungal in vitro activity.

### 2.2. Antibacterial Activity

#### 2.2.1. Bacteriostatic Activity

The antibacterial activity of TP10-NH_2_-based conjugates, as well as its constituents, was evaluated by microdilution assay in Mueller Hinton broth, MHB (Table 1). CIP and LVX exhibited a strong antibacterial effect, with the minimal inhibitory concentration (MIC) values in the 0.0125–0.8 (MIC_50_)/1.6 μM (MIC_90_) range. TP10-NH_2_ transportan displayed the highest activity against *Escherichia coli* and *Staphylococcus epidermidis*, with an MIC_90_ value equal to 1.6 μM, while the Gram-negative bacterium *Pseudomonas aeruginosa* appeared resistant (MIC > 100 μM). Slightly higher MIC values for TP10-NH_2_ against *Staphylococcus* *aureus* ATCC 25,923 and *E. coli* ATCC 25,922 (both 16 μg/mL, corresponding to ~7 μM) were obtained by Xie and co-workers [23]. The antibacterial activity of most of the novel conjugates of CIP and LVX with TP10-NH_2_ (**1**–**6)** was generally lower than that of the constituent fluoroquinolones. There are, however, some exceptions. Among the conjugates, the highest activity was found for CIP-S-S-TP10-NH_2_ (**3**), which displayed slightly better or similar activity against *S. epidermidis* and *E. coli* compared to CIP. Its activity against two other tested bacteria was somewhat lower though. The activity of the conjugate with CIP directly attached to TP10-NH_2_ (**2**) was slightly lower (MIC values in the 0.8–3.125 μM range). Further reduction of the antimicrobial activity was observed when CIP was linked to TP10-NH_2_ by the disulfide bridge between two Cys residues (compound **6**). Undoubtedly, the lowest activity was noted for the CIP-CH_2_CO-TP10-NH_2_ (**1**) conjugate. The LVX-based conjugates **4** and **5** were less active than the CIP-based **1**, **2**, **3,** and **6**. LVX-TP10-NH_2_ (**4**) demonstrated moderate activity against Gram-positive bacteria (MIC_90_ = 1.6 μM), but Gram-negative bacteria were much less susceptible. The conjugate LVXC-S-S-CTP10-NH_2_ (**5**), having both components connected via the disulfide bridge formed by cysteine residues, was more active against the Gram-negative bacteria than **4**, but its activity against Gram-positive bacteria was lower.

Further investigation was conducted to determine the antibacterial effect of the equimolar mixtures of CIP or LVX with TP10-NH_2_. The results are listed in Table S2 with the compared values for the mixture components. This data indicates that the fluoroquinolones and the peptide in combination do not act synergistically.

Additionally, for selected compounds, the aspect of the influence of salts present in the medium on antimicrobial activity was evaluated. For that purpose, antibacterial activity in MHB was compared to that in the MHB2 medium, supplemented with Ca(II), 20–25 mg/L, and Mg(II), 10–12.5 mg/L. Data shown in Table S3 indicate that the presence of salts strongly affected the antibacterial activity of TP10-NH_2_ but had very little, if any, influence on the activity of the conjugates. In the salt-enriched medium, the MIC values determined for TP10-NH_2_ were up to 40-fold higher than those found in MHB. The phenomenon of reduction of the growth inhibitory activity of antimicrobial peptides at a high salt concentration was previously evidenced by other researchers [24,25]. It may cause severe limitations for AMPs’ usage as therapeutic agents because of the risk of potential inactivation by salts present in the human body. We also observed a slight decrease of antibacterial activity in MHB2 for conjugates **1** and **6**. However, the MICs of conjugates **3** and **4** in MHB and MHB2 were identical or only slightly different.

#### 2.2.2. Long-Term Resistance Development

**Figure 2:**
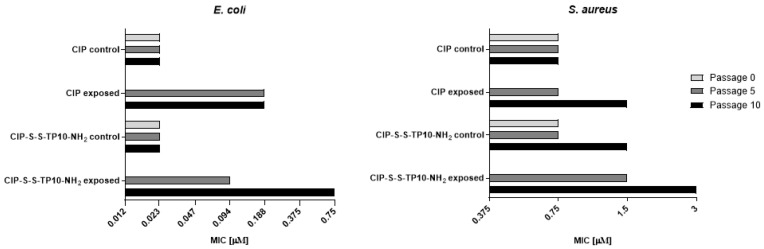
Possibility of resistance development for ciprofloksacin and CIP-S-S-TP10-NH_2_ (**3**) by *Escherichia coli* (**left**) and *Staphylococcus aureus* (**right**) after prolonged exposure to their action. Cells were passaged 10 times on Mueller Hinton agar plates with the addition of given compounds in the maximal concentrations allowing growth. MIC values were marked initially and after the 5th and the 10th passage and compared to the control. The mean values of three repeats are presented.

The *E. coli* ATCC 25,922 and *S. aureus* ATCC 29,213 cells were serially passaged (10 times) on Mueller Hinton agar (MHA) plates in the presence of CIP (0.012 and 0.1875 μM, respectively) or CIP-S-S-TP10-NH_2_ (**3**) (0.012 and 0.375 μM, respectively). The MIC values were determined under standard conditions against cells collected from colonies grown at the initial plate and plates after the 5th and the 10th passage. An analogous determination was performed for the control cells, passaged without exposure to antimicrobials. As shown in Figure 2, in the case of *E. coli* cells, the MIC values for CIP remained unchanged for control cells and increased 8-fold for cells after the 5th and the 10th passage. Similarly, MIC values for the conjugate **3** remained unchanged for the control cells, while for the cells exposed to compound **3**, increased only 4-fold after the 5th passage but 32-fold after the 10th one.

The *S. aureus* resistance to CIP and compound **3** (Figure 2) developed at lower rates than that of *E. coli* since only a 2-fold increase of the MIC values of both agents compared to the control was observed.

### 2.3. Bactericidal Effect

#### 2.3.1. Minimal Bactericidal Concentration

The minimal bactericidal concentration (MBC) was determined for TP10-NH_2_ as well as conjugates **3** and **5** (Table S4). The selected compounds exhibited bactericidal activity against four tested bacteria strains (concentrations ranging from 3.12 to 100 μM). The lowest MBC value was obtained for conjugate **3**. The conjugates **3** and **5** were superior to TP10-NH_2_ transportan for *E. coli*, *P. aeruginosa*, and *S. aureus*. However, no significant difference was noted for *B. subtilis*. The ratio of MBC to MIC_90_ for conjugate **5** was 2.0 for all tested bacteria strains.

#### 2.3.2. Kinetics of Bactericidal Effect

**Figure 3:**
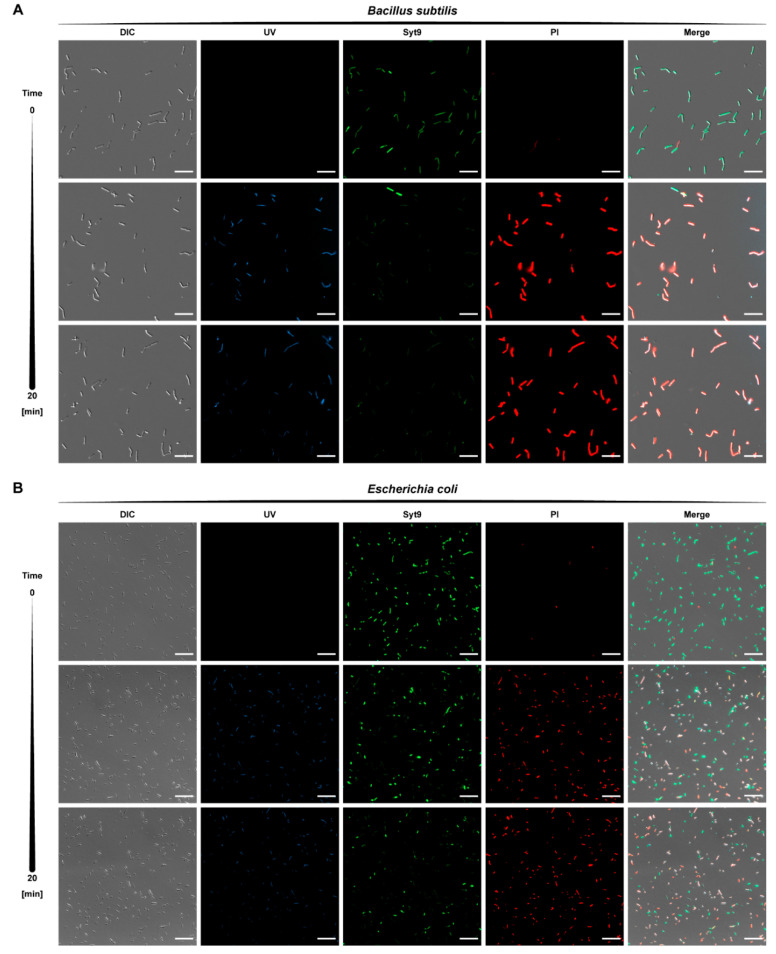
Death kinetics of (**A**) *B. subtilis* and (**B**) *E. coli* treated with conjugate **5** at Minimal Bactericidal Concentration followed over 20 min. Within the first 10 min of incubation, the compound penetrates the cells (blue) and disrupts the membrane, which allows the uptake of propidium iodide (PI, red), displacing DNA-bound Syto 9 (green). No morphological alterations were observed. Scale bars correspond to 20 μm.

As described before, fluoroquinolone conjugates with TP10-NH_2_ are active against both Gram-positive and Gram-negative bacteria, with MBCs reaching micromolar levels (Table S4). Furthermore, UV-induced fluorescence of fluoroquinolones allowed observation of conjugate localisation. Differential live-dead bacteria staining was performed for conjugate **5** at MBC (Figure 3). After 10 min of incubation with conjugate **5,** the green fluorescence (vital) was decreased with a simultaneous increase in red fluorescence (dead), a result of disruption of the membrane integrity marked by Syto 9 displacement by propidium iodide. Dead cells exhibited concurrent UV-inducible blue fluorescence emission (conjugate accumulation), illustrating the bactericidal properties of the conjugate **5**. No significant discrepancies in the results were noted between Gram-positive (*B. subtilis*) and Gram-negative (*E. coli*) representatives.

Additionally, real-time transmitted light and fluorescence microscopy were performed for conjugate **5** at MBC. Conjugate **5** was able to penetrate four tested bacteria strains and decrease their mobility (Videos S1–S4). Conjugate **5** accumulated in the cells within the first minute after addition. After 15 min, the majority of the cells showed a considerable decrease in mobility and fluorescence corresponding to the conjugate. In this experiment, the compound penetrated the cells faster in the case of the Gram-negative than Gram-positive bacteria, thereby affecting their mobility.

### 2.4. Antifungal Activity

The antifungal in vitro activity of CIP, LVX, TP10-NH2, and conjugates **1**–**6** was evaluated against six strains of the *Candida* genus, including three reference strains, the *C. albicans* mutant lacking genes coding for peptide permeases, and two clinical *C. albicans* strains Gu4 and Gu5. Gu4 is a Fluconazole (FLU)-sensitive isolate obtained from an early infection episode, while Gu5 is the corresponding FLU-resistant isolate obtained from a later episode in the same patient treated with FLU. The Gu5 isolate is resistant to FLU, due to the FLU-induced overexpression of genes encoding the drug efflux pump encoded by the CDR1/CDR2 genes, and Gu4 exhibits a basal expression of these genes [26].

The MIC values obtained are shown in Table 2. No growth inhibition of all strains by the fluoroquinolones CIP and LVX at concentrations up to 200 μM was observed, while TP10-NH_2_ exhibited poor activity exclusively against *C. albicans* (MIC = 100 μM). The activity of the conjugates **1**–**6** was generally higher than that of their components The highest activity was observed for CIP conjugates **2**, **3**, and **5** (MICs in the 6.25–100 μM range). The *C. glabrata* strain was resistant to the action of all the tested compounds. The MIC values of TP10-NH_2_ and conjugates against the *C. albicans* peptide permeases-deficient mutant were the same or slightly lower than those against the wild-type strains. This finding suggests that these compounds are not transported through the cell membrane by peptide permeases. The MIC values of all conjugates against *C. albicans* and *C. krusei* were in most cases lower than those of the established antifungal drug, Fluconazole. The FLU-resistant clinical Gu5 strain was sensitive to conjugates **1**–**6** at the same level like its FLU-sensitive counterpart Gu4.

### 2.5. Molecular Mechanism of Antifungal Activity

#### 2.5.1. Cellular Uptake

**Figure 4:**
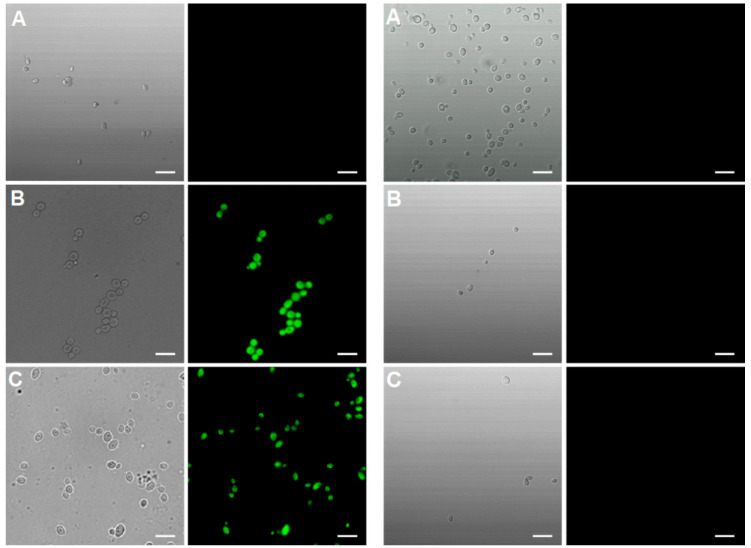
Uptake and accumulation of (**A**) CIP(***Cf***); (**B**) TP10(***Cf***)-NH_2_; and (**C**) CIP-CH_2_CO-TP10(***Cf***)-NH_2_ (**7**) in *Candida albicans* cells–left microphotography column. Right microphotography column-uptake and accumulation of (**A**) CIP(***Cf***); (**B**) TP10(***Cf***)-NH_2_; and (**C**) CIP-CH_2_CO-TP10(***Cf***)-NH_2_ (**7**) in *Candida glabrata* cells. The fluorescent signal of the compounds is shown in green. Cells were suspended in phosphate-buffered saline and incubated in the presence of fluorescent probes (40 μM) for 30 min. Differential interference contrast and fluorescence microscopy. Scale bars correspond to 10 μm.

**Figure 5:**
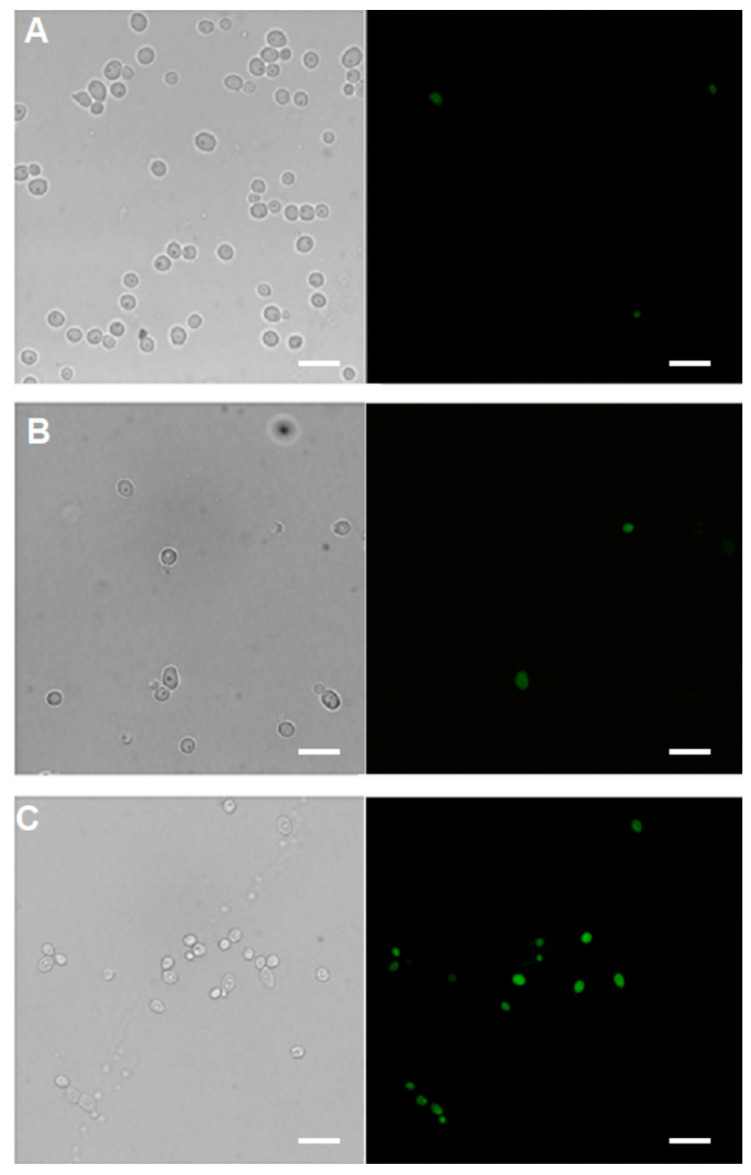
Kinetics of CIP-CH_2_CO-TP10(***Cf***)-NH_2_ (**7**) uptake by *Candida albicans* cells. Pictures were taken after (**A**) 0, (**B**) 15, (**C**) 30 min. Scale bars correspond to 10 μm.

**Figure 6:**
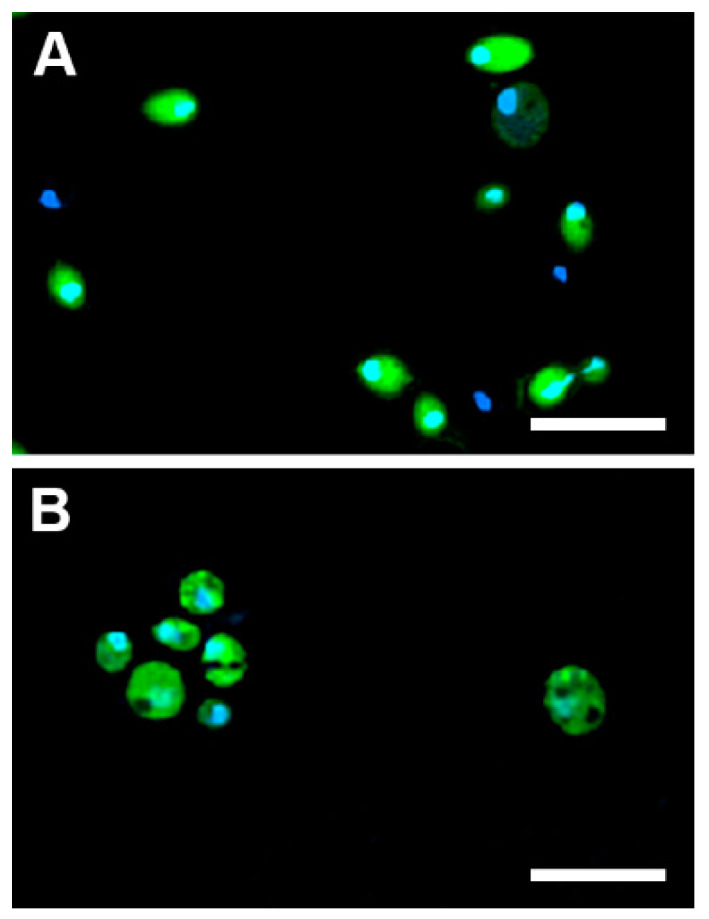
Confocal images of *C. albicans* cells loaded with (**A**) CIP-CH_2_CO-TP10(***Cf***)-NH_2_ (**7**) and (**B**) TP10(***Cf***)-NH_2_. The fluorescent signal of the compounds is shown in green. Hoechst 33,342 was used to stain nuclei. Scale bars correspond to 10 μm.

Figure 4 clearly demonstrates the qualitative difference in the uptake of the analysed compounds to *C. albicans* and *C. glabrata.* Both TP10(***Cf***)-NH_2_ and CIP-CH_2_CO-TP10(***Cf***)-NH_2_ (**7**) were transported to and accumulated in *Candida albicans* cells, while CIP(***Cf***) did not enter these cells. On the other hand, none of the fluorescent probes were taken up by *Candida glabrata* (Figure 4). These results provide a rational explanation for the lack of antifungal activity of CIP and LVX, as well as for the apparent resistance of *Candida glabrata* to fluoroquinolones, TP10-NH_2_, and the conjugates. The uptake of CIP-CH_2_CO-TP10(***Cf***)-NH_2_ (**7**) was time dependent, as evidenced in Figure 5. The confocal images shown in Figure 6 confirm that CIP-CH_2_CO-TP10(***Cf***)-NH_2_ and TP10(***Cf***)-NH_2_ taken up by *C. albicans* cells are uniformly distributed in the cytoplasm.

#### 2.5.2. Inhibition of Yeast DNA Topoisomerase II

Ciprofloxacin, levofloxacin, and other analogue quinolones are well-known inhibitors of bacterial gyrase. Studies showed that fluoroquinolones inhibit this enzyme through DNA binding rather than direct enzyme inhibition [27,28]. Fluoroquinolones are also able to inhibit eukaryotic DNA topoisomerases I and II. However, complete inhibition of eukaryotic enzymes requires a much higher concentration of those drugs [29].

**Figure 7:**
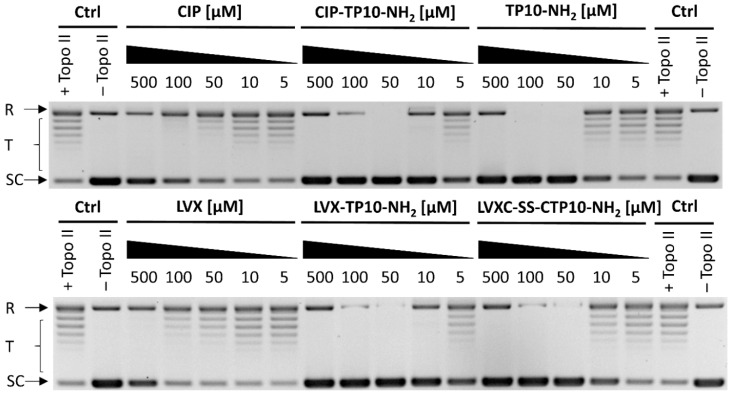
In vitro inhibition of the catalytic activity of pure type II yeast topoisomerase enzyme by fluoroquinolones and their peptide conjugates. R: relaxed DNA, T: DNA topomers, SC: supercoiled DNA.

In order to check whether tested conjugates inhibit yeast DNA topoisomerase II in the same manner as CIP and LVX alone, the supercoiled plasmid DNA relaxation assay mediated by yeast DNA topoisomerase II was performed. As shown in Figure 7, TP10-NH_2_ and the studied conjugates were able to inhibit yeast topoisomerase II in a concentration-dependent manner. TP10-NH_2_ was able to abolish enzyme activity at a concentration of 50 µM entirely. CIP-TP10-NH_2_ (**2**) and LVX-TP10-NH_2_ (**4**) completely inhibited topoisomerase II activity at 10 µM, whereas LVX-C-S-S-CTP10-NH_2_ (**5**) at 50 µM. Under the experimental conditions, CIP and LVX displayed residual activity at the highest used concentration of 500 µM.

**Figure 8:**
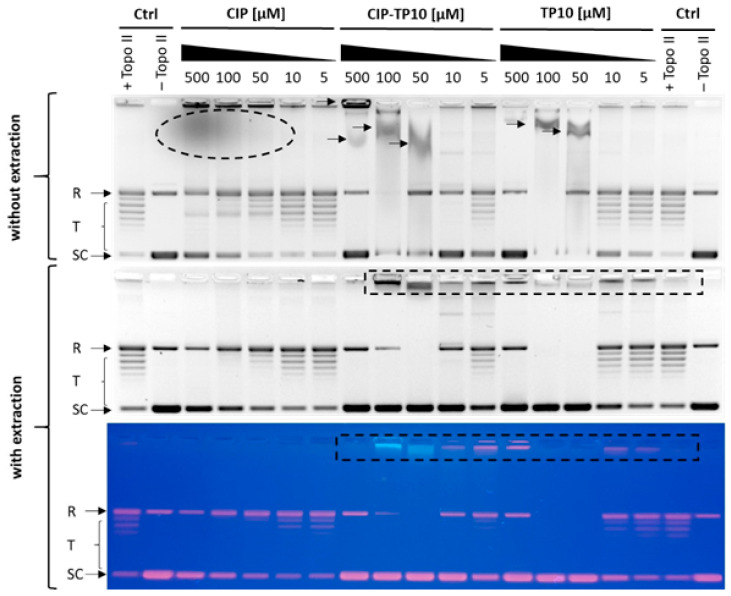
Results of parallel electrophoretic separation of samples from one experiment with the extraction (lower panel) and without extraction step (upper panel). Unbound CIP fluorescence was highlighted with a dotted ellipse. Weak DNA–peptide interactions are marked with arrows. Strong DNA–peptide interactions are highlighted with a dotted rectangle. The bottom colour picture shows comigrating DNA–peptide bands with mixed fluorescence: red from DNA (EtBr) and blue fluorescence from CIP-TP10-NH_2_ conjugate (highlighted with a dotted rectangle). R: relaxed DNA, T: DNA topomers, SC: supercoiled DNA.

Both CIP and LVX are fluorescent and thus can be visualised in agarose gel after electrophoresis under UV light without any additional staining. The results of parallel electrophoretic separation of samples from one experiment are shown in Figure 8. Electrophoresis of the relaxation assay samples without previous chloroform/isoamyl extraction step revealed that TP10-NH_2_ and CIP-TP10-NH_2_ (**2**) might interact with DNA in at least two distinct manners. Firstly, we observed relatively weak peptide–DNA interaction (indicated by arrow), which can be destroyed by the chloroform/isoamyl alcohol extraction step. The observed bands are distorted and characterised by a delay in gel mobility compared to the band from the relaxed form of the plasmid DNA. Secondly, we observed relatively strong peptide–DNA interaction (indicated by the dotted rectangle), which cannot be removed by extraction. The bands are sharp and exhibit poor gel mobility. On the colour photographs presented on the bottom panel of Figure 7, we observed the co-migration band containing DNA (red fluorescence from EtBr) and conjugate (blue fluorescence from CIP-TP10-NH_2_ (**2**)). Similar results were obtained for LVX (Figure S2).

We postulate that TP10-NH_2_ and its conjugates inhibit DNA topoisomerase II in two different ways. At low concentrations, 5–100 µM, the peptide and the conjugate most probably act as direct inhibitors of the enzyme by interacting with a distorted DNA intermediate arising during topoisomerase II-mediated catalysis. This assumption is supported by the observation of vanished bands from mostly unwound (R: relaxed) form of plasmid DNA at the 100 and 50 µM peptide concentrations tested and retention of slow-migrating bands after the organic-solvent extraction step. However, we did not observe any slow-migrating bands remaining after extraction at the highest used concentration of TP10-NH_2_ and its conjugates. We assume that at 500 µM, the formation of enzyme–DNA intermediate is not possible, probably due to the direct sequestration of negatively charged DNA by positively charged peptide or other factors, i.e., possible changes in pH, direct enzyme inactivation before the DNA binding step.

### 2.6. Mammalian Toxicity

#### 2.6.1. Cytotoxicity

The cytotoxicity of CIP, LVX, and conjugates **1**–**6** was evaluated on three mammalian cell lines: Epithelial, adherent kidney proximal tubule cells isolated from kidney of 3- to 4-week-old male pigs (LLC-PK1), human embryonic kidney cells (HEK 293), and human liver cancer cell line (Hep G2). Fluoroquinolones CIP and LVX were less cytotoxic than TP10-NH_2_ and the conjugates (Table 3). Among the conjugates, LVXC-S-S-CTP10-NH_2_ (**5**) was the least cytotoxic. In the case of Hep G2 cells, the half-maximal inhibitory concentration (IC_50_) value for compound **5** was nine times higher than that for CIP-TP10-NH_2_ (**2**), which was found to be the most cytotoxic. In the case of HEK 293 and LLC-PK1 cells, the IC_50_ values of **5** were nearly four times higher. Two other CIP-based conjugates CIP-CH_2_CO-TP10-NH_2_ (**1**) and CIP-S-S-TP10-NH_2_ (**3**) were comparably cytotoxic to all three cell lines and similarly to **2** had lower IC_50_ values than **5**. An approach to attach CIP to TP10-NH_2_ via the Cys residue appeared to be beneficial in terms of the potential cytotoxicity, as the IC_50_ values for CIPC-S-S-CTP10-NH_2_ (**6**) were more than two times higher than for **2** in the case of two cell lines. Additionally, we noted that the cancer cells Hep G2 were less sensitive to the action of the given compounds than the non-cancer cells (HEK 293 and LLC-PK1).

There is little doubt that the observed cytotoxicity of conjugates was due to the effect of the TP10-NH_2_ component, since the IC_50_ values of all but the compound **5** conjugates and the peptide were at a similar level, while CIP and LVX were not cytotoxic at all.

#### 2.6.2. Hemolysis

**Figure 9:**
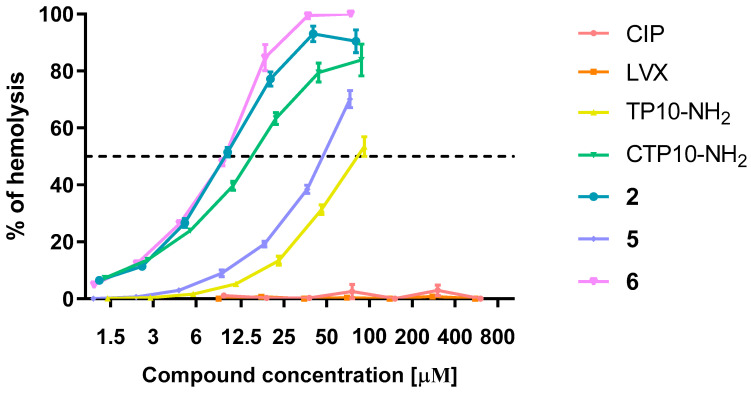
The hemolytic activity of the selected conjugates and their constituents.

Additionally, to evaluate the potential mammalian toxicity of the tested compounds, the hemolysis assay was performed. The hemolytic effect of the tested compounds on human red blood cells is shown in Figure 9. TP10-NH_2_ is known to cause hemolysis, perhaps due to the high content of hydrophobic amino acid residues (62%) [30,31]; however, in our case, 50% hemolysis by this peptide was observed at nearly 200 μg/mL (~92 μM). Even though antimicrobial drugs CIP and LVX do not cause a hemolytic effect, the conjugates were hemolytic, with MHC_50_ values (minimum hemolytic concentration that caused 50% hemolysis of human red blood cells) in the 25–140 μg/mL range. Furthermore, for all congeners, the hemolytic activity was higher than for TP10-NH_2_ alone. A similar effect was recently described by others for TP10-NH_2_-derived antimalarial conjugates [32]. The LVX congener **5** was the least hemolytic among the conjugates, with IC_50_ = 140 μg/mL (~51 μM), which is in line with its lowest cytotoxicity (Table 3). The most hemolytic was CIP-TP10-NH_2_ (**2**), with MHC_50_ = 25 μg/mL (~10 μM). The MHC_50_ values of the conjugates were much higher than the MIC_50_ values against bacteria (Table 1) but lower than the MIC_50_ values against yeasts (Table 2).

#### 2.6.3. ROS Generation

**Figure 10:**
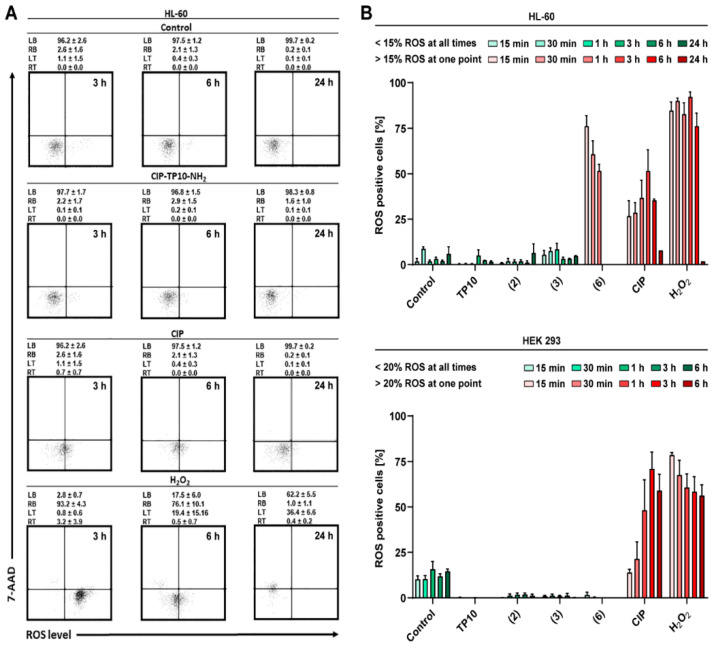
Reactive oxygen species (ROS) generation activity of TP10-NH2 and selected conjugates. (**A**) Dot plot representation of selected data. Cells were counterstained with 7-AAD as membrane integrity marker. (**B**) Comparison of the ROS generating effect on two cell lines. The values shown are means ± SD (*n* = 5).

The results of reactive oxygen species (ROS) generation are shown in Figure 10. In the positive controls treated with H_2_O_2_, the fraction of ROS-positive cells exceeded 75%. In reference samples treated with CIP, the ROS positive fraction exceeded 50%. Strikingly, in HL-60, a biologically significant effect was observed almost immediately (after 15 min of incubation). The same was noted after 1 h in the HEK 293 cell line. On the other hand, for a **2** and **3,** no ROS generation was observed. Similarly, conjugates **4** and **5** generated ROS at the low level (less than 15% for HL-60 and less than 20% for HEK 293) (manuscript in preparation). Surprisingly, the two tested cell lines drastically differed in response to conjugate **6**. For HL-60, the critical level of ROS was noted after 15 min of incubation. On the contrary, in HEK 293, the conjugate did not induce a significant level of ROS. The fact that the action of most of the synthesised conjugates results in negligible ROS generation is advantageous, suggesting low cardio- and hepatotoxicity potential, which stands in strike contrast with bare antibacterial fluoroquinolones [33,34,35].

### 2.7. Selectivity in Relation to Mammalian Cells

#### 2.7.1. Antibacterial

The bacteriostatic selectivity index (BSI) values were calculated (BSI = IC_50_/MIC_90_ or MHC_50_/MIC_90_) and are presented in Table S5. For the majority of the tested compounds, BSI values were positive (>1) ranging from 1.2 to 662.8. Conjugate **3** exhibited exceptionally high BSI values for *E. coli*, averaging at 534.3. Even though the hemolytic activity of the conjugates was increased compared to bare fluoroquinolones, the related selectivity index values of the conjugates were still favourable.

Additionally, the bactericidal selectivity index (BcSI) values were calculated (BcSI = IC_50_/MBC or MHC_50_/MBC) and are presented in Table S6. Analogous to the BSI values, for the majority of the tested compounds, the BcSI values were positive (>1), ranging from 2.0 to 16.8. Particularly, the selectivity index values related to the hemolytic activity of the conjugate **5** were still positive.

#### 2.7.2. Antifungal

Positive mycostatic selectivity index values (MSI = IC_50_/MIC_90_ or MHC_50_/MIC_90_) were obtained for conjugate **5**, ranging from 1.2 to 4.4 (Table S7). The highest MSI values were noted for *Saccharomyces cerevisiae* and *Candida albicans*.

### 2.8. Summary

The results from all performed experiments were summarised in the form of a table (Table 4). With selectivity both for bacteria and yeast cells and no ROS generation activity, the conjugate 5 possesses the most favourable properties of all synthesised conjugates.

## 3. Materials and Methods

### 3.1. Solid-Phase Peptide Synthesis (SPPS)

Peptides were synthesised by a solid-phase approach using the Fmoc/Boc*^t^* chemistry at 50 μmole scale, applying a Prelude peptide synthesiser (Gyros Protein Technology, Inc., USA). A TentaGel S RAM resin (substitution 0.24 meq/g, Rapp Polymere, Germany) was used as a solid support, yielding, after cleavage, peptides with amide on their *C*-termini. Peptide chains were elongated in the consecutive cycles of deprotection and coupling. Deprotection was performed with 20% piperidine in *N*,*N*-dimethylformamide (DMF) and the peptide chain elongation was affected using a reaction mixture composed of *N*,*N*,*N*′,*N*′-tetramethyl-*O*-(benzotriazol-1-yl)uroniumtetrafluoroborate (TBTU), 1-hydroxybenzotriazole (HOBt), *N*-methylmorpholine (NMM), and a three-fold molar excess of each *N*-α-Fmoc protected amino acid derivative (GL Biochem, Shanghai, China). After completing the synthesis, peptides were cleaved from the resin and the protecting groups were removed in a one-step procedure using a mixture of TFA:phenol:triisopropylsilane:H_2_O (88:5:2:5, v/v/v/v). The crude peptides were purified on Beckman Gold System (Beckman, Miami, FL, USA) equipped with an RP Supelco Discovery BIO, Wide Pore C8, 10 mm column (10 × 250 mm, Sigma Aldrich, St. Louis, MO, USA) or by PLC 2050 Gilson HPLC with Gilson Glider Prep. Software (Gilson, France), equipped with Grace Vydac C18 (218TP) HPLC column (22 × 250 mm, 10 µm, 300 Å, Resolution Systems). The solvent systems were 0.1% trifluoroacetic acid (TFA) in water (A) and 0.1% TFA in 80% acetonitrile in water (B). Different linear gradients were applied (flow rate 5.6 or 20 mLmin^−1^, monitored at 226 nm). The purities of the synthesised peptides were checked with an HPLC Pro Star system (Varian, Australia) and use of a Kinetex 5 μm XB-C18 100 Å column (4.6 × 150 mm, Phenomenex^®^, Torrance, CA, USA). The solvent system was as described above. A linear gradient from 10% to 90% B for 40 min, flow rate 1 mLmin^−1^, monitored at 226 nm was used. All described peptides had purities of at least 95%. In order to confirm the correctness of the molecular masses of the synthesised peptides, mass spectrometry analysis was carried out by a MALDI-TOF/TOF, Autoflex MAX spectrometer, (Bruker, Billerica, MA, USA) with an α-cyano-4-hydroxycinnamic acid (CCA) and/or 2,5-dihydroxybenzoic acid (DHB) matrix.

### 3.2. Synthesis of Levofloxacin and Ciprofloxacin-Based Conjugates

In the case of CIP-TP10-NH_2_ (**2**) and LVX-TP10-NH_2_ (**4**), Boc-CIP (obtained by the reaction of CIP with di-tert-butyldicarbonate in the presence of NaOH) and LVX were manually attached to the peptidyl-resin. An equimolar mixture of *N*,*N*′-diisopropylcarbodiimide (DIPCI), *N*,*N*′-diisopropylethylamine, and LVX or Boc-CIP (3 equiv. each) were dissolved in DMF/DCM (*v*/*v*; 1/1) and added to the SPPS vessel with peptidyl-resin and allowed to stir for 90 min. The procedure was repeated until the chloranil test gave a negative result. In order to obtain CIP-CH_2_CO-TP10-NH_2_ (**1**), ciprofloxacin was coupled to the peptide via the submonomeric approach as described previously [36]. After assembling the peptide chain and the removal of the Fmoc group, bromoacetic acid (5 equiv.) was attached to the peptidyl-resin using DIPCI (5 equiv.) in DMF. The procedure was repeated twice. Next, 1.5 equiv. of both ciprofloxacin and triethylamine in DCM/DMF was used to attach the constituent antimicrobial. The reaction mixture was stirring for 24 h at room temperature. In the case of CIP-S-S-TP10-NH_2_ (**3**), a solution of Lomant’s reagent (3,3′-dithiodipropionic acid di(*N*-hydroxysuccinimide ester))–DSP (1.2 equiv.) was dissolved in DMF, added to the SPPS vessel with peptidyl resin, and shaken overnight. The procedure was repeated twice. Ciprofloxacin and triethylamine (1.5 equiv. each) were dissolved in DMF/DCM and added to peptidyl-resin with coupled DSP. The reaction mixture was stirred for another 24 h at room temperature. All conjugates were detached from the solid support together with the removal of side-chain protection groups and purified as described above. To obtain LVXC-S-S-CTP10-NH_2_ (**5**), the disulfide bridge between LVX-Cys (described below in Section 3.2.1) and CTP10-NH_2_ was formed. Briefly, CTP10-NH_2_ (46 mg, 0.02 mmol) was dissolved in 5 mL of DMF and LVX-Cys(Npys) (12 mg, 0.02 mmol) was added. The mixture was stirred for 4 h at room temperature. The reaction was monitored by analytical HPLC. After 4 h, the solvent was removed in vacuo, and the conjugate was purified by semi-preparative HPLC; yield 20%; the product was characterised by HPLC and MALDI-TOF MS.

In the case of CIPC-S-S-CTP10-NH_2_ **(6)**, the disulfide bridge was formed during the reaction of CTP10-NH_2_ and Cys(Npys)-CIP (see below Section 3.2.2.) as described above for **5**. The total yield was 19 %. The product was characterised by HPLC and MALDI-TOF MS.

#### 3.2.1. Synthesis of LVX-Cys(Npys)

LVX-Cys(Npys) was obtained in two steps. Firstly, levofloxacin-*N*-hydroxysuccinimidyl ester (LVX-NHS) was synthesised. The esterification reaction between the carboxylic group of LVX (119 mg, 0.33 mmol) and the hydroxyl group of *N*-hydroxysuccinimide (38 mg, 0.33 mmol) was carried out using *N*,*N*′-dicyclohexylcarbodiimide (DCC, 68 mg, 0.33 mmol) in DMF (6 mL). The solution was stirred overnight, and then DMF was evaporated under vacuum. The obtained residue was extracted with 10 mL of ethyl acetate and washed five times with water (5mL, portion each time) and twice with ice-cold saturated NaHCO_3_ (5 mL). Then, the washed extract was concentrated under vacuum and left overnight to crystallise at room temperature. The product isolated by recrystallisation in EtOH gave LVX-NHS in a 65% yield. In the next step, Cys(Npys) (34 mg, 0.12 mmol) and DIPEA (21 µL, 0.12 mmol) dissolved in 5 mL of DMF were added to the solution of LVX-NHS (55 mg, 0.12 mmol) (pH was adjusted to 8-9 by NaOH). The reaction mixture was stirred overnight. The solvent was removed in vacuo and the product was treated several times with ether to wash out the unreacted ester. The remaining material was dissolved in water and lyophilised (yield 83%, 89 mg).

#### 3.2.2. Synthesis of Cys(Npys)-CIP

Boc-Cys(Npys)-OSu was obtained in the reaction of Boc-Cys(Npys)-OH with *N*-hydroxysuccinimide in the presence of *N*,*N*′-dicyclohexylcarbodiimide (DCC) as the coupling reagent (see above). In the next step, CIP (66 mg, 0.2 mmol) in 5 mL of DMF/DCM (3/2; v/v) was added to the solution of Boc-Cys(Npys)-OSu (94 mg, 0.2 mmol) in 3 mL of DMF. The reaction mixture was stirred overnight. The solvent was removed in vacuo, and the product was treated several times with ether (containing 10% DCM) to wash out the unreacted ester. The Boc-protecting group was removed by TFA (5 mL, 15 min at room temperature). The solution was concentrated in vacuo, and then the remaining material was dissolved in water and lyophilised (yield 92%, 128 mg).

Synthesis of fluorescently labelled compounds

In order to obtain fluorescein-labelled ciprofloxacin, 5(6)-carboxyfluorescein *N*-succinimidyl ester was prepared. The solution of 5(6)-carboxyfluorescein and *N*-hydroxysuccinimide (0.2 mmol each) was stirred in the presence of *N*,*N*′-dicyclohexylcarbodiimide (0.2 mmol) in DMF/DCM, first for 15 min at 0 °C and then for 3 h at RT. In the next step, solvents were evaporated in vacuo, and the residue was crystallised from methanol. The yellowish precipitate was drained over reduced pressure and washed with methanol (yield 76%). Then, 5(6)-carboxyfluorescein *N*-succinimidyl ester and ciprofloxacin (0.05 mmol each) were suspended in DMF/DCM and allowed to stir overnight. The yellow precipitate was filtrated over reduced pressure, suspended in water, lyophilised, and analysed as described above (yield 87%).

### 3.3. Synthesis of Fluorescently Labelled Compounds

In order to attach fluoresceine (***Cf***) to TP10-NH_2_, an analogue of TP10-NH_2_ containing Lys(Mtt) (Mtt stands for 4-methyltrityl) in position 12 instead of Ala was synthesised on a solid phase. After completing the synthesis, the Mtt protection of an *ε*-amino group of Lys was removed by 3-min incubations in 1.8% TFA in DCM until reaction completion, indicated by the disappearance of the yellow color (Mtt cation) in the drained deprotection solution. Then, the fluorophore was attached manually using 3 equiv. of 5(6)-carboxyfluorescein (Novabiochem, Merck, Darmstadt, Germany), HATU, HOAt, and DIPEA (molar ratio 1:1:1:2). The procedure was repeated three times. Next, TP10(***Cf***)-NH_2_ was cleaved from half of the peptidyl-resin, and the crude peptide was purified as described above. The other half of the peptidyl-resin was used to obtain the labelled CIP-CH_2_CO-TP10(***Cf***)-NH_2_ (**7**). The synthesis was performed by manual attachment of bromoacetic acid to the peptidyl-resin, and then CIP was coupled via the sub-monomeric approach. Finally, the labelled conjugate was removed from the solid support. The crude product was purified by semi-preparative HPLC, and characterised by HPLC and MS analysis.

### 3.4. Microorganism Strains and Growth Conditions

The following strains were used for antimicrobial activity tests: *C. albicans* ATCC 10231, *C. albicans* SC5314, *C. glabrata* DSM 11226, *C. krusei* DSM 6128, *C. albicans* opt1-opt5Δptr2Δptr22Δ, *E.coli* ATCC 25922, *P. aeruginosa* ATCC 27853, *S. aureus* ATCC 29213, and *S. epidermidis* ATCC 12228. *C. albicans* Gu4, and Gu5 clinical isolates [26] were gifts from Joachim Morschhäuser, Würzburg, Germany. The yeast strains were maintained on YPD agar plates (1% (*w*/*v*) yeast extract, 2% (*w*/*v*) peptone, 2% (*w*/*v*) glucose, % (*w*/*v*) agar) in 30 °C and bacterial strains on LA (Luria-Bertani agar; 1% (*w*/*v*) tryptone, 1 % (*w*/*v*) NaCl, 0.% (*w*/*v*) yeast extract, 2% agar) plates in 37 °C for 16–24 h.

### 3.5. Stability Testing

Cell culture of *S. aureus* ATCC 25,923 was refreshed on solid LA overnight (37 °C). The next day, cell suspensions were prepared in LB liquid medium, and after about 2 to 3 h (logarithmic growth phase), CIP-S-S-TP10-NH2 (**3**) was added at concentrations of 16 μgmL^−1^ in a volume of 1 mL. The cultures were continued overnight (37 °C, 180 rpm). The next day, 1 mL of cell cultures was transferred to 2-mL tubes and centrifuged (12,000 rpm, 1 min, 4 °C) (from this moment, the samples were kept on ice). The supernatant was discarded and the pellet was suspended in 0.5 mL of PBS. Zirconia beads (A&A Biotechnology, Gdynia, Poland) and 350 μL of lysis buffer (Bioline) were added to the samples. The tubes were shaken on a BeadBeater device to break the cell wall (3 × 20 s). The suspension was transferred to new tubes and 8.5 μL of Protease Inhibitor Cocktail (100 × concentrated, Fermentas, Waltham, MA, USA) was added. The tubes were centrifuged (12,000 rpm, 20 min, 4°C). The supernatant was transferred to new tubes and stored at −20 °C until the LC-MS analysis. Before MS analyses, the samples were desalted using ZipTip^®^ C18 Pipette Tips (Merck Millipore Ltd., Burlington, MA, USA) according to the manufacturer’s protocol. LC-MS experiments were performed on a Qtof lcms9030 spectrometer (Shimadzu, Japan) equipped with an electropress ion source, LC Nexera X2 module with autosampler. Samples were dissolved in water and analysed on an XB C18 Aeris Peptide column (Phenomenex) o (100 mm × 2.1 mm); 3.6 μm bead diameter. The LC system was operated with mobile phase: solvent A: 0.1% formic acid in H_2_O and solvent B: 0.1% formic acid in MeCN. Samples were separated with a linear gradient (optimised for the best separation of the analysed samples), maintained at a flow rate of 0.2 mLmin^−1^. The injection volume was between 0.1 and 0.5 μL.

### 3.6. Antibacterial Activity Assay

The antibacterial activity (bacteriostatic: MIC and bactericidal: MBC) of the tested compounds was determined in MHB (Mueller Hinton Broth, Sigma Aldrich, St. Louis, MO, USA) using a serial two-fold dilution method in 96-well microtiter plates according to CLSI recommendations, described in M07-A10 document. The exact conditions have been described previously [9]. All experiments were performed in three replicates. The MIC_90_ and MIC_50_ values were defined as the concentrations of compounds tested that caused a 90% or 50% reduction of growth, respectively, comparing to the drug-free control.

### 3.7. Determination of Resistance Induction Potential

*E. coli* ATCC 25,922 or *S. aureus* ATCC 29,213 cells were taken from −80 °C and freshly grown on Mueller Hinton Agar (MHA) plates. The MIC values for CIP and CIP-S-S-TP10-NH_2_ (**3**) were determined. The cells (10^8^ CFU) were grown on MHA plates containing one of the tested compounds at several concentrations equal or slightly higher than the MIC values determined as described in Section 2.7. The highest concentration at which the cells were able to grow (MIC-10^8^) was selected for the rest of the experiment. Cells (10^8^ cells per plate) were incubated on MHA plates in the presence or absence (control) of the tested compound at MIC-10^8^ and passaged 10 times at 1- to 3-day intervals. MHA plates with the addition of the tested compound were freshly prepared every time before the passage. After the 5th and the 10th passage, cells from colonies grown on the plates were collected for determination of MIC values.

### 3.8. Antifungal Activity Assay

The antifungal activity of the tested compounds was assessed in 96-well microtiter plates, in buffered RPMI-1640 medium according to the CLSI recommendations (M27-A3 document, Clinical Laboratory Standards Institute 2008). The lowest concentration that prevented the growth of microorganisms was assigned as MIC, while the lowest drug concentration that caused 90% or 50% inhibition of growth compared to the drug-free control was assigned as MIC_50_ and MIC_50_, respectively. All experiments were performed in three replicates.

### 3.9. Cytotoxicity Assay

Cytotoxicity was assessed against three mammalian cell lines: LLC-PK1, Hep G2, and HEK 293. Multiwell (96–well) plates were seeded at 7000 cells/well in Medium 199 supplemented with 7.5% FBS, MEM Eagle’s medium supplemented with 10% FBS, or DMEM medium supplemented with 10% FBS, respectively. All media were supplemented with L-glutamine and antibiotics (penicillin/streptomycin). Cells were allowed to attach overnight. Drugs were dissolved in cell culture medium and added to wells in 100-µL aliquots of 2× concentrated solutions, in triplicates. To control wells, 100 µL of the medium were added. Cells were incubated with the studied compounds for 72 h at 37 °C and 95%/5% or 90%/10% (HEK 293 cells) CO_2_ atmosphere. Then, 20-µL aliquots of MTT solution in PBS (4 mg/mL) were added to all wells and plates were incubated further for 3 h in 37 °C. Formazan crystals formed were dissolved in 100 µL of DMSO and the absorbance of the solutions was measured using a multiwell plate reader (Spark, Tecan, Männedorf, Switzerland) at λ = 540 nm. Cytotoxicity was determined compared to the drug-free control. All experiments were performed in triplicates.

### 3.10. Measurement of ROS Generation

ROS generation activity was evaluated as described previously [37]. Briefly, HEK 293 cells were seeded on 35-mm Petri dishes in the amount of 15,000 cells per plate for 24 h in DMEM medium. Compounds were added at final concentrations: CIP-TP10-NH_2_ (**2**) 5 µM, and CIP 500 µM. HL-60 cells were seeded on 35-mm Petri dishes in the amount of 25,000 cells/5 mL in RPMI 1640 medium and compounds were added at final concentrations: CIP-TP10-NH_2_ (**2**) 2.5 μM, and CIP 100 µM. Positive controls contained 250 µM H_2_O_2_, while negative controls were without any additions. After 0.5, 2.5, 5.5, and 24 h of incubation at 37 °C, the CM-H2DCFDA molecular probe (Thermo Fischer Scientific, Waltham, USA) was added to the plates and incubation was continued for another 0.5 h. The HEK 293 cells were detached with 0.05% trypsin solution in PBS and HBSS (Hank’s Balanced Salt Solution) and suspended in fresh DMEM medium. All samples were additionally stained with 7-AAD (7-aminoactinomycin D, 0.8 µg/mL) and immediately analysed with a Guava easyCyte flow cytometer (Merc, Burlington, USA). The determinations were performed in triplicates.

### 3.11. Determination of the Haemolytic Potential

Blood samples were kindly provided by the Regional Center for Blood Donation and Blood Treatment in Gdańsk. Erythrocytes were prepared as described previously [38]. The hemolysis assays were performed in 96-well plates. The tested compounds were serially diluted in PBS in the concentration range 200–3.125 μg/mL and 100-μL aliquots were poured into wells. 1% Triton X-100 solution, 100 μL, was used as a positive control and 100 μL of the PBS solution served as a negative control. Erythrocyte suspensions in PBS (100 μL) were added to the wells, and plates were incubated at 37 °C for 1 h and then centrifuged (500× *g*, 5 min). Supernatants collected (100 μL) were transferred to new microtitration plates. Absorbance at each well was measured at 540 nm. All experiments were performed in biological triplicates.

### 3.12. Monitoring of the Cellular Uptake

Overnight cultures of *C. albicans* ATCC 10,231 cells in YBG medium were centrifuged at 5000 rpm for 3 min, and the cells were rinsed with phosphate-buffered saline (PBS). Cells were suspended in PBS and incubated with fluorescein-labelled CIP, TP10-NH_2_, or conjugate (**7**) (20 µg/mL) at 30 °C for 15 min. If appropriate, one drop of nuclear-staining dye Hoechst 33,342 solution (1 µg/mL) was added to the sample. Cell suspensions were centrifuged (5000 rpm, 3 min, room temperature), rinsed three times with PBS buffer, and cells were suspended in PBS buffer. Cellular fluorescence was visualised using a lens 63×, at λ_ex_/λ_em_ = 485 nm/520 nm for fluorescein and λ_ex_/λ_em_ = 350 nm/461 nm for Hoechst 33342, using an Olympus BX60 epifluorescence microscope (Olympus, Tokyo, Japan) or LSM 800 T-PMT confocal microscope (Carl Zeiss AG, Oberkochen, Germany) with a CCD camera. Images were acquired and processed with ZEN Blue software.

### 3.13. Inhibition of DNA Relaxation Mediated by DNA Topoisomerase II

A yeast DNA topoisomerase II Relaxation Assay Kit was purchased from Inspiralis (Norwich, UK) and assays were performed according to the manufacturer’s procedure. Briefly, the reaction mixture contained 500 ng of pBR322 DNA in reaction buffer (10 mM Tris-HCl, pH 7.9, 5 mM MgCl_2_, 100 mM KCl, 2% (*v*/*v*) glycerol, 1 mM ATP) as well as the studied compounds dissolved and diluted in dd H_2_O at the indicated concentrations. The reaction was initiated by the addition of topoisomerase II and allowed to proceed at 30 °C for 30 min. Reactions were terminated by the addition of 5 µL of the loading buffer (NEB; #B7024). The studied compounds were extracted from the reaction mixtures on vortex (for 30 s) with 30 µL of chloroform/isoamyl alcohol solution (24:1; *v*/*v*). After centrifugation (3 min, 20,000× *g*), half of the upper aqueous phases were separated in 1% agarose gels at 90 V for 4 h in TBE buffer (90 mM Tris-base, 70 mM boric acid, 1 mM EDTA, pH 8). Gels were stained with 1 μg/mL ethidium bromide (EtBr) for 15 min to visualise DNA and unbound EtBr was removed by washing gel in 1 mM MgSO_4_ solution in dd H_2_O for 15 min. Gels were photographed under UV illumination with a ESSENTIAL V6 (UVITEC, Cambridge, UK).

### 3.14. Bacterial Viability Assay

Overnight bacteria cultures at 37 °C were diluted and grown up to OD_600_ 0.2. Cultures were centrifuged at 7000 rpm for 3 min, and the cells were washed with 0.9% NaCl and suspended in 0.9% NaCl. The tested compound was added at a concentration equal to the MBC value. Equal aliquots of 3.34 mM Syto 9 and 20 mM propidium iodide were mixed, and 1 μL was added to 300 μL of cell suspension. Then, 3–5 μL of the samples were spotted on 0.8% agarose pads in 0.9% NaCl. Microphotographs were acquired using a Zeiss Axio Observer microscope with a CCD camera. Images were processed in ZEN Blue 2.6 software.

## 4. Conclusions

Conjugation of CIP and LVX with TP10-NH_2_ markedly changed the biological properties of these drugs. Particularly, unlike the mother fluoroquinolones, their conjugates with the cell-penetrating peptide exhibited antifungal activity and mammalian cytotoxicity. The latter seems to be at least in part due to the intrinsic cytoplasmic membrane disruption activity of TP10-NH_2_, which was demonstrated in hemolytic studies. The earlier reports concerning the mammalian cytotoxicity of TP10 were confusing since no toxicity of this CPP against HeLa cells was observed [19]. In contrast, in another study, substantial membrane toxicity (measured by the lactate dehydrogenase leakage) against three cell lines and some hemolytic effect was noted [29,32,39]. On the other hand, the antifungal growth inhibitory effect of conjugates observed by us was clearly related to their facilitated uptake by sensitive yeast cells. All CIP, LVX, TP10-NH_2_, and their conjugates were found to be inhibitors of yeast type II DNA topoisomerase. CIP and LVX appeared as much weaker inhibitors of this enzyme than their conjugates with TP10-NH_2_. However, no similar relationship was found for the antifungal activity of cleavable (**3**, **5**, and **6**, possibly releasing a free drug intracellularly) and non-cleavable (**2**, **4**) conjugates. Therefore, topoisomerase II may not be a primary target of these compounds.

Conjugation with TP10-NH_2_ mostly lowered the antibacterial activity of the fluoroquinolones tested, although in the case of CIP-S-S-TP10-NH_2_ (**3**), this effect was negligible. It should be noted, however, that the conjugates **2**, **3**, and **5** demonstrated a relatively strong growth inhibitory effect against *Pseudomonas aeruginosa*, i.e., bacterium that appeared completely resistant to TP10-NH_2_.

The antifungal activity and mammalian cytotoxicity of redox-sensitive [10] **3** and **5** were very similar to that of their redox-resistant analogues **2** and **4**. On the other hand, the activity of cleavable **3** against *S. epidermidis*, *P. aeruginosa*, and especially against *E. coli* was better than that of non-cleavable **2**. It seems, therefore, that intracellular CIP release from its conjugate with TP10-NH_2_ facilitates the interaction of the fluoroquinolone with bacterial gyrase, while in eukaryotic cells, conjugate cleavage is not a prerequisite for effective interaction with a molecular target.

Although the antifungal effect has not been observed for all conjugates and all fungal cell lines, the observed phenomenon is, in our opinion, pretty interesting. Since the antifungal effect was much more robust in conjugates where TP10-NH_2_ was linked to the fluoroquinolone by a disulfide bond, it seems likely that the antifungal activity may be due to the interaction of parental drugs with DNA and nuclear enzymes, following proteolytic cleavage of the conjugate inside the cell. Additionally, the ligand covalently bound with TP10-NH_2_ may disrupt the membrane [40]. Therefore, the modification of fluoroquinolone’s action by linkage with TP10-NH_2_ is strictly a result of the conjugation. As we were able to show a proof of concept of expanding antibacterial drug action by antifungal activity for clinically established compounds (originally lacking this activity), it suggests a future perspective for the use of cell-penetrating peptides in clinical treatment. Additionally, we found an unusual mechanism of action, including both topoisomerase II inhibition and a membrane effect. Such observations suggest, however, a next step towards acquiring a possible antifungal drug candidate. This is a conjugation of CIP or LVX to a CPP exhibiting optimal parameters of mammalian cell toxicity. Alternatively, it is worth exploring the linking of TP10-NH_2_ with other antimicrobial drugs.

We were able to show a proof of concept that conjugation of CPP to an established antibacterial drug may result in the expansion of the activity spectrum. In particular, fluoroquinolones CIP and LVX conjugated to TP10-NH_2_ peptide acquired antifungal activity. Notably, this activity against *C. albicans* and *C. krusei* was slightly better than that of the known antifungal drug Fluconazole. Moreover, the clinical strain of *C. albicans* resistant to FLU due to the FLU-induced overexpression of Cdr1p and Cdr2p drug efflux pumps remained sensitive to conjugates **1**–**6**. This finding suggests that the conjugates are not good substrates for Cdr1p and Cdr2p. The cytotoxic potential of the conjugates, while interesting within the scope of anticancer approaches, reduces their potential for a safe use to address non-neoplastic conditions, e.g., fungal (and other microbial) infections. This actually agrees with previous promising reports on CP–drug conjugates. Their vast majority addresses potential anticancer applications, which is probably related to the fact that there is increasing evidence (also supported by this work) on the cytotoxicity and hemolytic activity of most such conjugates.

Interestingly, during our studies, we discovered the antileukemic activity of the studied conjugates (manuscript in preparation). The antileukemic activity added upon antibacterial, as well as novel antifungal activity, may prove advantageous for leukaemia treatment. Specifically, for patients receiving allogeneic blood marrow transplantation (allo-BMT), autologous blood marrow transplantation (auto-BMT), or peripheral blood stem cell transplants (PBSCT)s, bacterial infection remains one of the leading causes of morbidity and mortality (with an incidence ranging from 18.6% to 43.6%) despite early aggressive antimicrobial therapy [41,42]. The gold standard therapy regimens consist of either levofloxacin (LVX) or ciprofloxacin (CIP), exhibiting negligible anticancer activity [43,44,45].

Future investigations will be focused on the understanding of the fundamental molecular mechanism of action of the tested conjugates. Currently, we are performing computer simulation studies for selected conjugates to examine their interaction with the cell membrane, including the possibility of aggregation and formation of intra-membrane channels as well as interaction with DNA after proteolysis. We would also like to move to ex vivo studies on patient-derived cell lines.

## Figures and Tables

**Table 1:** Antibacterial activity of TP10-NH_2_ conjugates and its constituents in Mueller Hinton broth (MHB).

Compound	MIC [μM]							
	Gram (+)				Gram (-)			
	*S. aureus* ATCC 29213		*S. epidermidis* ATCC 12228		*E. coli* ATCC 25922		*P. aeruginosa* ATCC 27853	
	MIC_90_	MIC_50_	MIC_90_	MIC_50_	MIC_90_	MIC_50_	MIC_90_	MIC_50_
CIP	0.2	0.2	1.6	0.8	0.0125	<0.0125	0.4	0.4
LVX	0.4	0.4	1.6	0.8	0.025	0.025	0.8	0.8
TP10-NH_2_	3.125	3.125	1.6	1.6	1.6	1.6	>100	>100
**1**	12.5	12.5	12.5	6.25	25	25	>100	>100
**2**	0.8	0.8	1.6	1.6	1.6	1.6	3.25	3.25
**3**	0.8	0.8	0.8	0.8	0.025	0.0125	1.6	0.8
**4**	1.6	1.6	1.6	1.6	50	50	100	100
**5**	6.25	6.25	3.125	3.125	3.125	3.125	25	12.5
**6**	3.125	3.125	6.25	6.25	6.25	6.25	>100	>100

**Table 2:** The antifungal activity of CIP, LVX, TP10-NH_2_, and conjugates **1**–**6**.

Compound	MIC [μM]							
	*C. albicans* SC 5314		*C. albicans* Δopt1Δopt5Δptr2Δptr22		*C. albicans* Gu4	*C. albicans* Gu5	*C. glabrata* DSM 11226	*C. krusei* DSM 6128
	MIC_90_	MIC_50_	MIC_90_	MIC_50_	MIC_50_	MIC_50_	MIC_50_	MIC_50_
CIP	>200	>200	>200	>200	>200	>200	>200	>200
LVX	>200	>200	>200	>200	>200	>200	>200	>200
TP10-NH_2_	100	100	100	100	100	100	>200	>200
**1**	100	50	200	100	50	50	>200	50
**2**	12.5	12.5	12.5	6.25	25	25	>200	50
**3**	25	6.25	25	12.5	6.25	12.5	>200	25
**4**	100	50	100	100	25	25	>200	100
**5**	25	12.5	25	12.5	12.5	12.5	>200	50
**6**	50	50	50	50	25	25	>200	>200
FLU	100	50	100	50	50	>200 (1600)	100	50

**Table 3:** In vitro cytotoxicity (IC_50_ ± SD (μM)) of selected conjugates and their constituents toward human embryonic kidney cells, G2 human liver cancer cells, and old male pig kidney cells.

Compound	IC_50_ ± SD [μM]		
	HEK 293	Hep G2	LLC-PK1
CIP	>200	>200	>200
LVX	>200	>200	>200
TP10-NH_2_	25.08 ± 0.86	32.12 ± 1.65	22.73 ± 5.65
CTP10-NH_2_	19.47 ± 2.93	59.97 ±5.75	51.60 ± 8.66
**1**	10.81 ± 1.87	21.70 ± 2.18	17.79 ±1.93
**2**	10.42 ± 1.39	11.46 ± 2.03	27.06 ± 1.82
**3**	13.11 ± 1.22	19.03 ± 2.08	16.83 ± 2.62
**4**	22.54 ± 9.70	72.58 ± 12.95	52.92 ± 8.15
**5**	41.34 ± 1.10	101.5 ± 4.90	104.70 ± 15.62
**6**	20.84 ± 10.91	37.71 ± 2.84	21.33 ± 1.33

**Table 4:** Summary data of TP10-NH_2_ and its conjugates’ antimicrobial activity, mammalian toxicity, and molecular interactions.

	Antimicrobial Activity					
	Antibacterial Selectivity		Antifungal Selectivity			
Compound	HEK 293 LLC-PK1 for *E. coli*	HEK 293 LLC-PK1 for Gram +	HEK 293 LLC-PK1 for *C. albicans*	HEK 293 LLC-PK1 for *C. krusei*	HEK 293 LLC-PK1 for *ΔC. albicans*	HEK 293 LLC-PK1 for *C. glabrata*
TP10-NH_2_	++	++	-	-	-	-
**1**	-	+	-	-	-	-
**2**	++	++	-	-	+	-
**3**	++	++	-	-	-	-
**4**	++	++	-	-	-	-
**5**	++	++	+	+	+	-
**6**	-	+	-	+	-	-
	**Mammalian Toxicity**					
	**ROS**		**Hemolysis Selectivity**			
	**HL-60**	**HEK 293**	**HL-60**		**HEK 293**	
TP10-NH_2_	++	++	-		-	
CTP10-NH_2_	N/D	N/D	+		++	
**2**	++	++	+		+	
**3**	+	++	N/D		-	
**4**	+	++	N/D		-	
**5**	++	++	-		+	
**6**	-	++	+		+	
	**Molecular Interactions**					
	**Topoisomerase II Inhibition**		**Cellular Uptake**			
			**Bacterial**		**Fungal**	
			**Gram -**	**Gram +**	*C. albicans*	*C. glabrata*
					**Wall**	**Nucleus**
TP10-NH_2_	N/D		N/D	N/D	+	-
**3**	+		++	+	N/D	N/D
**4**	++		++	+	N/D	N/D
**5**	+		++	+	N/D	N/D
**7**	N/D		N/D	N/D	+	-

Antimicrobial Activity: ++ SI > 10, + SI > 1; Cellular Uptake ++ within 10 min, + within 1 h; - not detected within 2 h; Topoisomerase inhibition ++ IC_50_ < 10 µM, + IC_50_ < 50 µM; SI ++ SI > 4, + SI > 1; ROS ++ <10% positive cells in 24 h, + <15% positive cells in 24 h; N/D not determined.

## Supplementary Materials

Supplementary Materials can be found at https://www.mdpi.com/1422-0067/21/13/4696/s1.

## Abbreviations

AMP	antimicrobial peptide
BcSI	Bactericidal Selectivity Index
BSI	Bacteriostatic Selectivity Index
CCA	α-cyano-4-hydroxycinnamic acid matrix
Cf	5(6)-carboxyfluorescein
CIP	ciprofloksacin
CPP	cell-penetrating peptide
DCC	*N*,*N*′-dicyclohexylcarbodiimide
DCM	dichloromethane
DHB	2,5-dihydroxybenzoic acid matrix
DIC	Differential Interference Contrast
DIPCI	*N*,*N*′-diisopropylocarbodiimide
DIPEA	*N*,*N*-diisopropylethylamine
DMF	*N*,*N*-dimethylformamide
EtBr	ethidium bromide
HATU	(1-[bis(dimethylamino)methylene]-1H-1,2,3-triazolo[4 ,5-b]pyridinium 3-oxide hexafluorophosphate
HEK293	human embryonic kidney cells
HeLa	human cervical cancer cells
HepG2	human liver cancer cells
HL-60	human myeloid leukemia cells
HOAt	1-hydroksy-7-azabenzotriazol
HOBt	1-hydroxybenzotriazole
IH_50_	Minimum Hemolytic Concentration
LLC-PK1	old male pig kidney cells
LVX	levofloxacin
LVX-NHS	levofloxacin-*N*-hydroxysuccinimidyl ester
MBC	Minimal Bactericidal Concentration
MDR	multidrug-resistance
MHA	Mueller Hinton Agar
MHB	Mueller Hinton Broth
MIC	Minimal Inhibitory Concentration
MSI	Mycostatic Selectivity Index
Mtt	4-methyltrityl
NMM	*N*-methylmorpholine
Npys	Npys
PBS	phosphate-buffered saline
ROS	reactive oxygen species
TAT	trans-activator protein
TBTU	*N*,*N*,*N*′,*N*′-tetramethyl-*O*-(benzotriazol-1-yl)uroniumtetrafluoroborate
TFA	trifluoroacetic acid
