# Supplementary material for: Conjugates of Ciprofloxacin and Levofloxacin with Cell-Penetrating Peptide Exhibit Antifungal Activity and Mammalian Cytotoxicity
Source: Int J Mol Sci. 2020 Jun 30;21(13):4696. doi: 10.3390/ijms21134696 (PMC7369900; doi:10.3390/ijms21134696)
Supplement: Supplementary file 1 [file ijms-21-04696-s001.zip › Supplementary_Materials-Rev.docx]

**Supplementary Materials**

**Materials and Methods**

*Bactericidal effect kinetics*

Overnight bacteria cultures at 37 °C were diluted and grown, up to OD_600_ 0.2. The tested compound was added at a concentration equal to the MBC value. 3-5 μL of the samples were spot on microscope slides and protected with a coverglass. Videos were acquired using Zeiss Axio Observer microscope with a CCD camera, processed in Zeiss ZEN Blue and edited in Adobe Premiere Pro CC software.

**Figures**

**
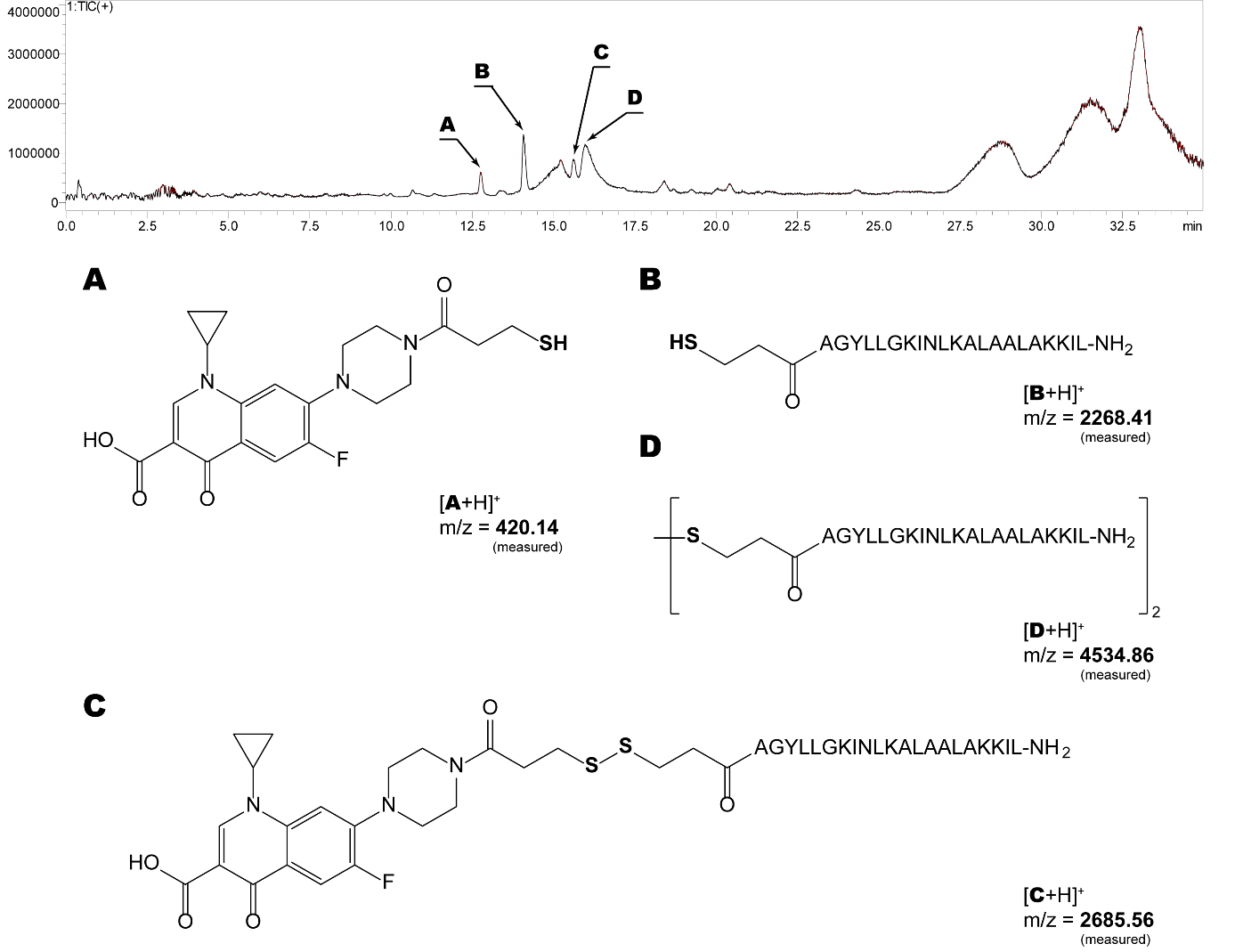
**

**Figure S1**. Mass Spectrometry analysis of *S. aureus* cell lysate after incubation with the conjugate **3**. Identified peaks were illustrated with structures of corresponding compounds.


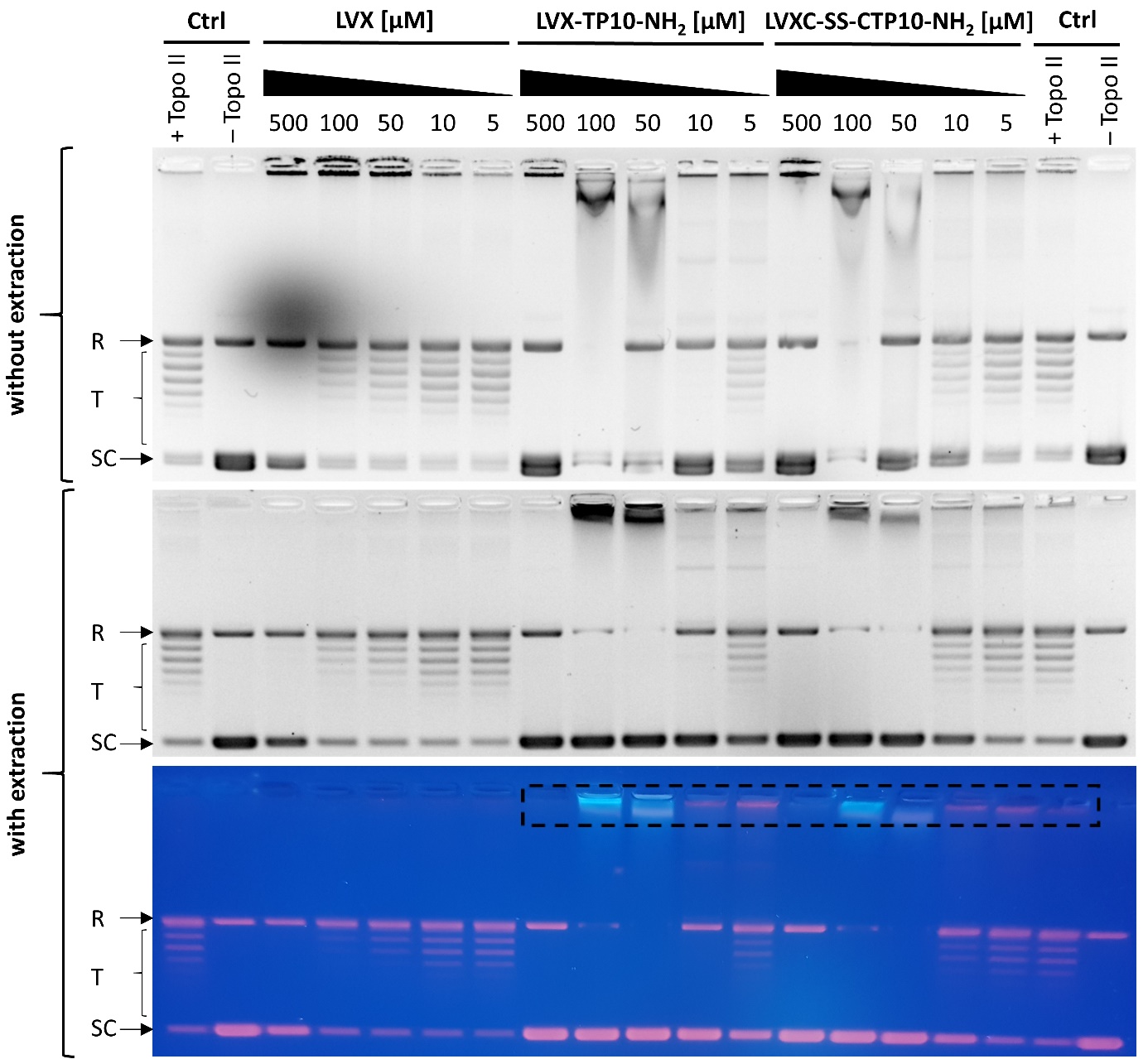


**Figure S2**. For levofloxacin and its conjugates, the results of parallel electrophoretic separation of samples from one experiment are shown. The bottom color pictures shows co-migrating DNA:peptide bands with mixed fluorescence: **red** from DNA (EtBr), **blue** from LVX-TP10-NH_2_ (**4**) and LVXC-SS-C-TP10-NH_2_ (**5**) conjugates highlighted with dotted rectangle.

**Tables**

**Table S1.** Physicochemical properties of the peptide conjugates and their components.

| **Compound** | **Calculated monoisotopic**  **MW** | **Measured accurate mass [M+H]^+^** | **t_R_ [min]** | **Yield**  **[%]** |
| --- | --- | --- | --- | --- |
| Cys-CIP | 434.10 | 435.2 | 10.6 | 92 |
| LVX-Cys | 464.15 | 465.1 | 11.0 | 89 |
| CIP(***Cf***) | 689.18 | 689.7 | 13.3 | 87 |
| TP10-NH_2_ | 2180.41 | 2181.8 | 27.0 | 80 |
| TP10(*Cf*)-NH_2_ | 2597.81 | 2599.1 | 25.9 | 50 |
| CTP10-NH_2_ | 2283.42 | 2285.5 | 24.1 | 75 |
| CIP-CH_2_CO-TP10-NH_2_ (**1**) | 2551.54 | 2554.6 | 30.1 | 54 |
| CIP-TP10-NH_2_ (**2**) | 2493.54 | 2495.8 | 25.3 | 85 |
| CIP-S-S-TP10-NH_2_ (**3**) | 2685.53 | 2689.2 | 27.8 | 41 |
| LVX-TP10-NH_2_ (**4**) | 2523.55 | 2525.9 | 24.9 | 62 |
| LVXC-S-S-CTP10-NH_2_ (**5**) | 2745.56 | 2749.2 | 20.4 | 25 |
| CIPC-S-S-CTP10-NH_2_ (**6**) | 2715.55 | 2719.7 | 22.3 | 19 |
| CIP-CH_2_CO-TP10(***Cf***)-NH_2_ (**7**) | 2969.61 | 2972.5 | 27.9 | 30 |

MW – Molecular Weight

**Table S2.** The activity of the individual components of the conjugates in equimolar mixtures.

| **Compound** | **MIC_90_ [μM]** | |
| --- | --- | --- |
|  | ***S. aureus*** | ***E. coli*** |
| CIP | 0.2 | 0.0125 |
| LVX | 0.4 | 0.025 |
| TP10-NH_2_ | 3.125 | 1.6 |
| CIP+TP10-NH_2_ | 0.2 | 0.0125 |
| LVX+TP10-NH_2_ | 0.4 | 0.025 |

**Table S3**. Antibacterial activity of TP10-NH_2_ conjugates and its constituents in Ca(II) and Mg(II) enriched MHB2.

| **Compound** | **MIC [μM]** | | | | | | | | |
| --- | --- | --- | --- | --- | --- | --- | --- | --- | --- |
|  | **Gram (+)** | | | | | **Gram (-)** | | | |
|  | *S. aureus*  **ATCC 29213** | | *S. epidermidis*  **ATCC 12228** | | *E. coli*  **ATCC 25922** | | | *P. aeruginosa*  **ATCC 27853** | |
|  | MIC_90_ | MIC_50_ | MIC_90_ | MIC_50_ | MIC_90_ | | MIC_50_ | MIC_90_ | MIC_50_ |
| TP10-NH_2_ | 25 | 25 | 25 | 25 | 50 | | 50 | >100 | >100 |
| **1** | 6.25 | 3.125 | 6.25 | 6.25 | 25 | | 12.5 | >100 | >100 |
| **3** | 0.8 | 0.8 | 0.8 | 0.8 | 0.025 | | 0.0125 | 0.8 | 0.8 |
| **4** | 1.6 | 1.6 | 1.6 | 1.6 | 50 | | 50 | >100 | >100 |
| **6** | 25 | 25 | 12.5 | 12.5 | 1.6 | | 1.6 | 50 | 50 |

**Table S4.** Minimal Bactericidal Concentrations and MBC/MIC ratios for selected compounds.

| **Compound** | **Gram-negative** | | | | **Gram-positive** | | |
| --- | --- | --- | --- | --- | --- | --- | --- |
|  | *E. coli*  ATCC 25922 | | *P. aeruginosa*  ATCC 27853 | | *B. subtilis*  *ATCC* | *S. aureus*  *ATCC 29213* | |
|  | MBC [µM] | MBC / MIC_90_ | MBC  [µM] | MBC / MIC_90_ | MBC  [µM] | MBC  [µM] | MBC / MIC_90_ |
| TP10-NH_2_ | 12.5 | 7.8 | 100 | ND | 6.25 | 25 | 8 |
| **3** | 3.12 | >10 | 6.25 | 3.9 | 6.25 | 6.25 | 7.8 |
| **5** | 6.25 | 2 | 50 | 2 | 6.25 | 12.5 | 2 |

**Table S5.** Bacteriostatic selectivity of TP10-NH_2_ and its conjugates in relation to mammalian cell lines as well as hemolytic activity.

| **Compound** | **HEK 293**  for  *S. aureus*  *S. epidermidis*  *E. coli*  *P. aeruginosa* | **LLC-PK1**  for  *S. aureus*  *S. epidermidis*  *E. coli*  *P. aeruginosa* | **Hep G2**  for  *S. aureus*  *S. epidermidis*  *E. coli*  *P. aeruginosa* | **Hemolysis**  for  *S. aureus*  *S. epidermidis*  *E. coli*  *P. aeruginosa* |
| --- | --- | --- | --- | --- |
| TP10-NH_2_ | 7.8  15.3  15.3  <0.2 | 6.9  13.4  13.4  <0.2 | 10.0  19.4  19.4  <0.3 | 29.4  57.5  57.5  <0.9 |
| **3** | 12.5  12.5  400.4  6.3 | 20.7  20.7  662.8  10.4 | 16.9  16.9  539.6  8.4 | N/D |
| **4** | 12.4  12.4  0.4  0.2 | 27.6  27.6  0.9  0.4 | 38.5  38.5  1.2  0.6 | N/D |
| **5** | 9.5  19.0  19.0  1.2 | 17.5  35.0  35.0  2.2 | 16.2  32.5  32.5  2.0 | 10.6  21.1  21.1  1.3 |
| **6** | 11.2  5.6  5.6  <0.4 | 8.8  4.4  4.4  <0.3 | 11.8  5.9  5.9  <0.4 | 2.9  1.5  1.5  <0.1 |

N/D not determined

**Table S6.** Bactericidal selectivity of TP10-NH2 and selected conjugates in relation to mammalian cell lines and hemolytic activity.

| **Compound** | **HEK 293**  for  *E. coli*  *P. aeruginosa*  *B. subtilis*  *S. aureus* | **LLC-PK1**  for  *E. coli*  *P. aeruginosa*  *B. subtilis*  *S. aureus* | **Hep G2**  for  *E. coli*  *P. aeruginosa*  *B. subtilis*  *S. aureus* | **Hemolysis**  for  *E. coli*  *P. aeruginosa*  *B. subtilis*  *S. aureus* |
| --- | --- | --- | --- | --- |
| TP10-NH_2_ | 2.0  <1  4.0  1.0 | 1.8  <1  3.6  <1 | 2.6  <1  5.1  1.3 | 7.4  <1  14.7  3.7 |
| **3** | 4.2  2.1  2.1  2.1 | 5.4  2.7  2.7  2.7 | 6.1  3.0  3.0  3.0 | N/D |
| **5** | 6.6  <1  6.6  3.3 | 16.8  2.1  16.8  8.4 | 16.2  2.0  16.2  8.1 | 8.2  1.0  8.2  4.1 |

N/D not determined

**Table S7.** Mycostatic selectivity of conjugate **5** in relation to mammalian cell lines as well as hemolytic activity.

| **Compound** | **HEK 293**  for  *C. albicans*  *ΔC. albicans*  *C. krusei* | **LLC-PK1**  for  *C. albicans*  *ΔC. albicans*  *C. krusei* | **Hep G2**  for  *C. albicans*  *ΔC. albicans*  *C. krusei* | **Hemolysis**  for  *C. albicans*  *ΔC. albicans*  *C. krusei* |
| --- | --- | --- | --- | --- |
| **5** | 2.4  2.4  1.2 | 4.4  4.4  2.2 | 4.1  4.1  2.0 | 2.6  2.6  1.3 |
